# Nitrogen Levels Regulate Sugar Metabolism and Transport in the Shoot Tips of Crabapple Plants

**DOI:** 10.3389/fpls.2021.626149

**Published:** 2021-03-10

**Authors:** Lihua Zhang, Simin Sun, Yonghui Liang, Baiyun Li, Songya Ma, Zhengyang Wang, Baiquan Ma, Mingjun Li

**Affiliations:** State Key Laboratory of Crop Stress Biology for Arid Areas/Shaanxi Key Laboratory of Apple, College of Horticulture, Northwest A&F University, Yangling, China

**Keywords:** apple, nitrogen, sugar unloading, sugar metabolism, shoot tips

## Abstract

To comprehensively understand the responses of carbohydrate metabolism and transport to different levels of nitrogen supply in growing shoot tips of crabapple (*Malus hupehensis* Rehd), enzyme activities and related genes involved in the sugar metabolism pathway were investigated. The nitrogen and chlorophyll content of plants increased with increasing nitrogen supply. High nitrogen application increased the net photosynthesis rate and the growth rate of shoot tips but decreased the synthesis capability of sucrose and sorbitol in mature leaves. However, the shoot tips of plants under high-nitrogen treatment had higher contents of sucrose and sorbitol than did those under low-nitrogen treatment, while the activity of sucrose phosphate synthase and aldose-6-phosphate was increased and the transporters *MdSOT* and *MdSUT* were up-regulated. Moreover, the activities of enzymes involved in sucrose and hexose metabolism (including sucrose synthase, fructokinase, and hexokinase) were enhanced in the shoot tips of plants under high-nitrogen conditions, and the expression levels of *MdSUSY3* and *MdHK1* were significantly up-regulated. These findings indicate that a high nitrogen supply increases the metabolic capacity of assimilatory substances in shoot tips, accelerates the efficiency of sugar utilization and eventually leads to a rapid increase in the growth of shoot tips. Our results highlight that high nitrogen increases the capacity of sugar unloading and metabolic utilization in growing shoot tissues.

## Highlights

- High nitrogen increases the capacity of sugar unloading and metabolic utilization in the shoot tips.

## Introduction

As the core mineral nutrient element, nitrogen (N) influences the processes of photosynthesis, respiration, and carbohydrate and signal transport in plants by synthesizing active substances such as proteins, nucleic acids, hormones, chlorophyll, vitamins, and several metabolic enzymes and participates in material and energy metabolism in plant cells (Krapp, 2015). Plant growth is highly regulated by the nitrogen level in the soil. Nitrogen deficiency promotes root growth, while adequate nitrogen favors shoot growth (Dash et al., 2015; Huang et al., 2018). Accelerated growth is related to changes in the priority of soluble carbohydrates distribution to growing organs under different nitrogen conditions, while source leaf-derived carbohydrates provide a material basis and energy for plant growth and development (Wang and Ruan, 2016). Knowing the response of carbohydrate metabolism and distribution to nitrogen levels in different apple organs is necessary for understanding the relationship between carbohydrates and nitrogen and the mechanisms underlying plant adaptations to nitrogen levels in the environment.

Sink organs of plants must be supplied with carbohydrates provided by source leaves for metabolic maintenance, but whether sink organs obtain enough carbohydrates depends on the sink strength, which is associated with the capability to unload and metabolize translocated carbohydrates (Marcelis, 1996). In most plants, sucrose (Suc) is the main end-product of photosynthesis and the main form of carbohydrate transported over long distances in the phloem from photosynthetic source organs (leaves) to heterotrophic sink tissues (fruits, roots, and shoot tips), which is extremely critical to the allocation of resources (Rennie and Turgeon, 2009; Eom et al., 2012; Ruan, 2014). Sucrose can be unloaded from the phloem to sink organs via two pathways: symplastic or apoplastic pathways, and the function, type and developmental state of the sink affect the way that sugar exits the phloem (Oparka and Turgeon, 1999; Hennion et al., 2019). In other parts of shoot tips, sucrose can be transported passively, moving along the concentration gradient between cells through plasmodesmata or via active transport involving transporters against the concentration gradient, such as membrane-localized sucrose transporters (SUTs) and SWEET proteins (Riesmeier et al., 1992; Lemoine, 2000; Milne et al., 2018). Soluble sugars such as sucrose, glucose (Glc), and fructose (Fru) can be transported into the vacuole by corresponding transporters localized on the vacuolar membrane (SOT, SUT, TST, etc.), and increasing sugar concentrations in the vacuoles activate enzymes and genes associated with the sugar metabolism system, regulating the osmotic balance in different compartments of the cell (Hedrich et al., 2015).

Soluble carbohydrate contents in plants highly depend on the balance between their synthesis, degradation and export. The main enzymes associated with sucrose metabolism are sucrose phosphate synthase (SPS), sucrose synthase (SUSY), and invertase (INV; Stitt et al., 1988; Moriguchi et al., 1992; Sturm et al., 1999). Sucrose can be broken down into glucose and fructose by vacuolar acid invertase (AINV) or into fructose and UDP-glucose (UDPG) by SUSY (Ruan, 2014). Studies have shown that SUSY is more capable of catalyzing sucrose degradation than sucrose synthesis (Geigenberger and Stitt, 1993). Glucose and fructose are then phosphorylated to glucose-6-phosphate (G6P) and fructose-6-phosphate (F6P) by hexokinase (HK) and fructokinase (FRK), respectively. Conversely, the F6P and UDPG produced during sugar metabolism resynthesize sucrose via SPS and sucrose phosphate phosphatase (SPP, EC 3.1.3.24; Hoffmann-Thoma et al., 2010). Aldose-6-phosphate reductase (A6PR), which is involved in the anabolic metabolism of sorbitol (Sor), is mainly found in apple leaves and is rarely detected in sink tissues (Fan et al., 2009). There are two types of sorbitol dehydrogenase (SDH), the key enzyme involved in sorbitol degradation: NAD^+^-sorbitol dehydrogenase (NAD^+^-SDH) and NADP^+^-sorbitol dehydrogenase (NADP^+^-SDH). As a key enzyme for breaking down sorbitol NAD^+^-SDH, catalyzes the conversion of sorbitol to fructose, after which the product catalyzed by NADP^+^-SDH is glucose. Additionally, NAD^+^-SDH is also abundantly distributed in apple callus, roots, seedlings, and young leaves but presents low or undetectable activity in source organs such as mature leaves (Biancoa et al., 1999). Some studies have revealed that the activity and expression level of these enzymes involved in sugar metabolism are affected by levels of exogenously applied nitrogen, such as those in the leaves of *Amaranthus cruentus* (Terashima and Evans, 1988), grape (Eynard et al., 2000), and apple (Chen and Cheng, 2004).

Sugar provides carbon skeletons for the composition of various structural substances in plants, and carbon skeletons are required for the assimilation of inorganic nitrogen into amino acids, proteins, and nucleic acids. Thus, nitrogen uptake and utilization are highly correlated with sugar availability. The effect of nitrogen on carbon assimilation has been described in Rosaceae, including apple and other plant species. Nitrogen deficiency causes significant accumulation of unstructured carbohydrates in plant leaves (Paul and Driscoll, 1997; Geiger et al., 1999), and a low nitrogen supply can alter the assimilate distribution between various organs. During the delayed period of maize grain filling, low nitrogen supply promotes the distribution of recently assimilated photosynthetic products to shoots and roots and reduces the distribution to reproductive organs, but the concentration of sugar in the grain increases despite the decrease in assimilates delivered to the reproductive organs (Paponov and Engels, 2005). However, the study of Chen and Cheng (2004) on Gala apple plants showed that sorbitol, sucrose, glucose and fructose levels in the leaves decreased or remained the same with a decrease in nitrogen application level, probably because nitrogen deficiency directly affected the activity of RuBP carboxylase/oxygenase (Rubisco) and other enzymes associated with photosynthesis, rather than the inhibition of carbon assimilation through feedback on soluble carbohydrate accumulation. The assimilation rate of CO_2_ increases with the increasing amount of nitrogen applied but decreases when the nitrogen level exceeds a certain value (Paul and Driscoll, 1997). In addition, excessive applications of nitrogen fertilizer can result in a decrease in juice, soluble solids and Vc content in citrus fruit (Iwakiri and Nakahara, 1982; Feigenbaum et al., 1987) and can increase the susceptibility of the fruit to physiological diseases such as fungal disease (Bramlage et al., 1980) and cork spot disease (Curtis et al., 1990). However, knowledge is still limited about how nitrogen application levels affect soluble sugar metabolism to regulate carbon utilization and distribution in vegetative sink tissue, shoot tips of apple.

The purposes of this study were to explore how nitrogen regulates the carbon utilization and sink strength of vegetative organ shoot tips, which is helpful for understanding the relationship between nitrogen and carbon and lays a foundation for research on nutrient organ growth, nitrogen and carbon distribution regulation and the development of related techniques. We focused primarily on the regulation of gene expression and enzyme activity involved in the synthesis of sorbitol and sucrose and the subsequent conversion to sucrose and fructose in the shoot tips and leaves of apple plants under different nitrogen levels using a systematic expression analysis based on metabolic pathways. This analysis provides a relatively clear regulatory network of the various genes and enzymes participating in these metabolic pathways and the role of vital isoenzymes during the diversification of carbohydrate metabolism in the leaves and shoot tips of apple treated with different nitrogen levels.

## Materials and Methods

### Plant Materials

Two-year-old crabapple (*Malus hupehensis* Rehd) seedlings, which keep highly genetic stability due to apomixis, planted in plastic pots (28 cm × 21 cm) filled with clean sand were used in this study. All seedlings were grown in a garden at Northwest A&F University, Yangling (34°20’N, 108°24’E), Shaanxi, China. In May 2018, 90 seedlings displaying similar growth were selected for different nitrogen level treatments. The culture media were washed once a day for 5 days continuously to remove the remaining nutrients in the basin, then conducting treatment three days later. Except for the normal water supply, the nutrient solutions with different nitrogen concentrations were applied every 7 days during the experiment. Three nitrogen levels were designed and tested according to the methods of Huang et al. (2018): low nitrogen (LN), 0.3 mmol/L N; normal nitrogen (CN), 6 mmol/L N; and high nitrogen (HN), 30 mmol/L N. The nitrogen was supplied as NH_4_NO_3_. The 90 seedlings were separated into three groups in accordance with the three nitrogen treatments (each group included at least 12 plants as one biological replicate). Each pot was received 1.5 L of nutrient solution each time, and the test period lasted for 40 days.

### Photosynthetic Rate and Growth Changes

After 35 days of seedling treatment, 20 seedlings of each treatment displaying the same growth were randomly selected, and the net photosynthetic rate (Pn) of fully open leaves was measured between 9:00 am and 12:00 pm with a CIRAS-3 portable photosynthesis (PP Systems, Amesbury, MA, United States).

At the beginning of the treatment, 15 plants of each treatment were selected randomly to mark and measure the length of the first shoot tip as S1, and the length from the marker position to the latest first shoot tip was measured as S2 after treatment. The growth ratio of shoot during the treatment period was expressed as S2-S1/T (T represents treatment time).

At the end of the treatment, the shoot tips and leaves in the middle of each treated plant were randomly sampled, immediately frozen in liquid nitrogen and stored at –80°C until use. Moreover, 10 crabapple seedlings were randomly selected and removed from the media. The roots of each plant were washed, and all the white, new root samples of each plant were collected, placed on absorbent paper to absorb the surface-adhered moisture, weighed with an electronic balance and stored at −80°C. Another 5 plant roots of each treatment were put into Kraft paper envelopes and then dried to a constant weight.

### Measurement of Nitrogen Content

The total nitrogen content was obtained by the Kjeldahl method (Tang et al., 2012). Dried aboveground and underground samples were ground and sifted using an automatic grinding instrument (IKA, Staufen, Germany). Approximately 0.1 g of powder was transferred to a 100 mL tube, 5 mL of concentrated sulfuric acid was added, and the tube was heated at 360°C for 4–5 h. Approximately five drops of H_2_O_2_ were added with a glue dropper every 30 min until the solution was transparent and clear. After the mixture had cooled, the volume was brought to the scale line with distilled water. The total nitrogen content was measured by a high-resolution automatic chemical analyzer using a 5 mL solution.

### Measurement of Chlorophyll Content

Chlorophyll was extracted according to the methods of Wellburn (1994). Approximately 0.1 g of frozen apple leaves was weighed and placed into a 50 mL graduated centrifuge tube with a stopper, and 15 mL of 80% acetone was later added to the tube. The stopper was then inserted, and the tube was placed in a dark place overnight under vertically shaken 3–4 times. The next day, if all the samples had turned white, indicating that the chlorophyll had been completely extracted, 80% acetone was used to bring the volume to 40 mL. The supernatant was removed, and the absorbance was measured at 663, 645, and 470 nm, with 80% acetone used as a control.

### Measurement of Soluble Sugars

The contents of soluble sugars and sugar alcohols in the shoot tips and leaves were determined by gas chromatography-mass spectrometry (GC-MS) according to the methods of Li et al. (2016). The sugar was extracted from a 0.1 g sample in 1.4 mL of 75% (v/v) methanol, and 100 μL of ribitol was added as an internal standard. After fractionation of the non-polar metabolites into 100 mL of added chloroform, 2 and 50 μL polar phases of each sample were collected to analyze the highly abundant sucrose content and slightly lower abundance of hexose. The samples were dried in a vacuum concentrator to anhydrous condition, then derivatized with 40 μL methoxyamine hydrochloride and 60 μL N-methyl-N-trimethylsilyl-trifluoroace-tamide (MSTFA; Yang et al., 2018). After the extracted samples were bottled, the metabolites were determined via a GCMS-2010SE instrument (Shimadzu Corporation, Kyoto, Japan). Metabolites were identified via comparing and searching their counterparts from a mass spectral library based on gas chromatography (GC)/mass spectrometry (MS) system. Quantifications were according to standard curves from each metabolite and internal standard. And the starch was determined according to the method of Li et al. (2012).

### Assay of Enzyme Activities

Sucrose synthase, SPS, neutral invertase (NINV), A6PR, FRK, HK, and SDH extractions were performed as described by Moscatello et al. (2011) and Li et al. (2012). Approximately 0.5 g samples were rapidly vortexed with 2 mL of extract buffer containing 50 mM HEPES-KOH (pH 8.0), 5 mM MgCl_2_, 2 mM EDTA, 2 mM EGTA, 2 mM benzyl (benzamidine), 2.5 mM dithiothreitol (DTT), 2 mM 6-aminohexanoic acid, 0.1 mM 4-(2-amino) benzenesulfonyl fluoride hydrochloride (AEBSF), 0.1% BSA, 2% glycerol, 0.05% Triton X-100, and 2% polyvinylpolypyrrolidone (PVPP). The vortexed extract was centrifuged at 16,000 *g* at 4°C for 20 min, after which the supernatant was immediately desalted through a Sephadex G25 PD-10 column (GE Healthcare, Buckinghamshire, United Kingdom) using extraction buffer with a concentration of 50 mM HEPES-KOH (pH 7.4) but without BSA, Triton X-100 or PVPP as the equilibrium eluent. Desalted liquid was then used to analyze the content of soluble proteins and the activity of the enzymes.

The activity of NINV was assayed at 30°C for 30 min in a 500 μL reaction mixture that consisted of 100 μL of 400 mM acetic acid tripotassium phosphate buffer (pH 7.5), 50 μL of 1 M sucrose, 250 μL of H_2_O and 100 μL of desalted enzyme extraction solution (as a blank). The assay was stopped by boiling for 5 min after adding 500 μL of 3,5-dinitrosalicylic acid (DNS) chromogenic agent. The mixture was then cooled to room temperature, after which the absorption at 540 nm was measured. DNS was added to the boiled, inactivated enzyme extract before incubation, which served as a control. The enzyme activity is represented as the amount of enzyme used to produce 1 μmol glucose/h.

Sucrose phosphate synthase activity was measured according to the methods of Chen and Cheng (2004), with some modifications. Briefly, the assay mixture (70 μL) consisted of 50 mM HEPES-KOH (pH 7.5), 15 mM MgCl_2_, 1 mM EDTA, 4 mM F6P, 14 mM G6P, 16 mM UDPG, and 35 μL of desalted enzyme extract. The reaction was carried out at 37°C for 30 min followed by boiling in water for 10 min after the addition of 70 μL of 1 N NaOH. The cells were then incubated at 80°C for 8 min after the addition of 750 μL of 30% HCl and 250 μL of 1% resorcinol. NaOH was added to the boiled, inactivated enzyme extract before incubation as a control, and 100 μL of the reaction mixture was utilized to measure the absorptivity at 480 nm via a spectrophotometric assay.

For SUSY, assays were conducted at 30°C for 30 min in a 500 μL mixture that consisted of 80 mM MES (pH 5.5), 5 mM UDP, 100 mM sucrose and 100 μL of desalted enzyme extract, followed by boiling in water for 5 min after the addition of 0.5 mL of DNS chromogenic agent. We used inactivated enzyme solution and H_2_O as substitutes for fresh enzyme solution and UDP, respectively, in the blank sample.

Fructokinase and HK activities were assayed using improved methods of Renz and Stitt (1993). For FRK, a 1 mL assay mixture consisting of 50 mM Tris-HCl (pH 8.0), 4 mM MgCl_2_, 2.5 mM ATP, 0.33 mM NAD^+^, 1 U of G6P dehydrogenase (G6PDH), 60 μL of enzyme extract, 0.4 mM fructose, and 1 U phosphoglucoisomerase (PGI) was used. The mixture of HK did not contain any PGI, and 1 mM glucose was added rather than fructose. The change in NADH per minute was measured at 340 nm, which indicated the enzyme activity.

The procedure we used to detect A6PR activity was the same as that used by Negm and Loescher (1981). One milliliter of reaction mixture consisting of 0.1 M Tris-HCl (pH 9.0), 0.11 mM NADPH, 50 mM G6P, H_2_O and 25 μL of enzyme extract was ultimately used. The mixture was incubated at 27°C, and NADH reduction was measured at 340 nm. The A6PR activity was calculated according to the oxidation of 1 μmol NADPH/h.

The activity of SDH was measured according to the methods of Li et al. (2012). The assays were conducted at 37°C for 3 min in a 1 mL assay mixture that consisted of 300 mM sorbitol, 1 mM NAD^+^, 100 mM Tris-HCl (pH 9.6) and 200 μL of enzyme extract. NADH generation was measured at 340 nm after incubation with added sorbitol at 37°C for 3 min.

### RNA Extraction and qRT-PCR Assays

Total RNA was extracted from leaves and shoot tips using an RNAprep Plant Kit (Tiangen, Beijing, China) according to the manufacturer’s instructions. cDNA synthesis was performed using a PrimeScript^TM^ RT Reagent Kit and gDNA Eraser to minimize the possibility of genomic DNA contamination (TaKaRa, Dalian, China). Quantitative real-time polymerase chain reaction (qRT-PCR) analysis was conducted with a SYBR Green Premix Ex Taq Kit (Takara, Kyoto, Japan), and the reaction procedures were as follows: 95°C for 5 min followed by 40 cycles of 95°C for 2 s, 60°C for 20 s and 72°C for 20 s. Each reaction was repeated three times. The primer sequences used for qRT-PCR are listed in Table 1, and *MdActin* (CN938023) was used as an internal gene.

**TABLE 1:** Primers used in this study.

Gene	Forward primer sequence (5′–3′)	Reverse primer sequence (5′–3′)
*MdActin*	GGA CAG CGA GGA CAT TCA GC	CTG ACC CAT TCC AAC CAT AAC A
*MdA6PR*	GCA GTC GCT GAG AGA GTT TGG	GAT TCA CGA TAG TTT CCA TTT CGC
*MdSDH1*	ATA GAG GAA GTT GGG AGT GAG GT	TCT CCT GGA TGG ACA ACC TGA TT
*MdSDH2*	ACA CCA TCA AGA TCC TAC CTT TC	CAT TTC ATG GTC TTG AGG TAG TG
*MdSPS1*	AGT GTA GTA CTC AAG GGA GTT GG	TGC TCA TGG GGA AGG CTT TAC
*MdSPS6*	AGG TTC TGT TGA GTA TGG CAG TGA G	GTG CTT CAA GTG CCG CTG AGA
*MdSUSY1*	CTC AAG CGT GTT AAG CAA CAG	CTG AAT GGA ACA CGA AGA ATA TC
*MdSUSY3*	TTA TGG TTT CTG GAA GTA TGT GTC	GTC GAT GGC TTC AGG AAC AGA TT
*MdSUSY4*	GAC AGG AAC AAG CCA ATC ATC T	GCC TTC TCC TCA TTG TCC TTG
*MdSUSY5*	CAT GCC AAT TTC TTG CTG ACA C	CTT TCG TAT TGT CCT GGT CTG TC
*MdNINV1*	GTC CAT TGT TTC ATC ATT GGG TAC	GGT CGC TGC CAG TGA TTA TAC G
*MdNINV2*	GAG TTC CAG ACA GGC ATA AGG CT	CCA TCC GTC TAT CAA TCA TAC AGG
*MdCWINV1*	TAA CAA ATA TGT GGT GCT CCT CTG	ACC CTA GCT GTT ATG CAC GCC T
*MdCWINV2*	TTC AAA GCT AAA GGC AGA CAC G	GTA AAT CTA CAT CTA CAA AGC CAG C
*MdFRK1*	CTG CAT TGG CAT TTG TTA CAC TC	AAG ATG GGT TGA CCT GCA TGG T
*MdFRK2*	GTG GTG GAA TCC TTC GAG GTC AA	CAA ATT TCA GTA CCT CCC TCA ACC T
*MdFRK3*	AGA GTC AAG GGT ATG AAG GTA GAT G	CTC GTC CTG AAG CAA AGA AAG AT
*MdFRK4*	TCA GGA TGA GGA GGG GCT ACG AG	CTG CTT TAA GCA CTG GAG CAC AGC
*MdHK1*	CTG AAA GTG GTC GGG AGC AAA C	TGC ACG AGT GGC AAC TAT GTC G
*MdHK2*	TGG TGG ATT ATA CGA GCA TTA CA	TCC AGG GTA TTG TGA GTG AGA G
*MdHK3*	AGA TTG TGG CGG ATG TAT GTG AC	CAA CAG TCC TCT TGC CAA AAA TG
*MdSOT1*	GGT TAG AAT GAC GTG GGC AGT TAT	TCA TCA ACC TAT TCA CGG CCA C
*MdSUT1*	TGT TCC GTA TGC TTT GGT TTC TTC	AAT AGC TGA TCC CAA GGT CCA CT
*MdSUT2*	ACT CAC TAT GTA TCA GCA GAA T	TGA GAT GGC CTC CTT TAG ATT CT
*MdTST1*	TCG TCT ATT TCT GCG TCT TTG TC	CCG CTG CGT AAA TCC CAA AT
*MdTST2*	GTA CCG AAC GAT GGT CAG TTC TTC	TGA CTC CGG GTT CGA AAA GGT C

### Statistical Analysis

The data were subjected to analysis via ANOVA, where significant variance was accepted at *p*-value <0.05 using IBM SPSS Statistics 21. Graphs were constructed with SigmaPlot 13.0 software. The data are presented as the mean ± SD of three biological replicates.

## Results

### Nitrogen Content and Growth Changes Under Different Nitrogen Supplies

**FIGURE 1:**
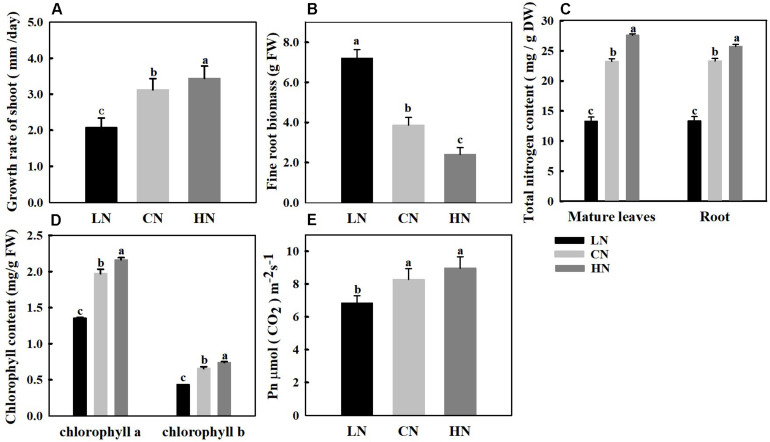
Changes in growth rate of shoot **(A)**, new fine root biomass **(B)**, total nitrogen content **(C)**, chlorophyll content **(D)**, and net photosynthesis (Pn) **(E)** in crabapple seedlings treated with different nitrogen levels. LN means low nitrogen level contains 0.3 mM nitrogen; CN means common nitrogen level contains 6 mM nitrogen; HN means high nitrogen level contains 30 mM nitrogen. Each value is mean ± SD for three biological replicate samples and each sample was a mixture of five plants. Bars labeled with different letters indicate significant difference between treatments.

Nitrogen levels affect plant growth, and the total nitrogen content directly reflects the nitrogen nutrient level of plants. To determine the effects of different nitrogen treatments on crabapple, growth changes of the shoots (Figure 1A) and roots (Figure 1B) and the total nitrogen content (Figure 1C) of treated plants were measured. The results showed that the total nitrogen content of the leaves and roots was approximately 23 mg/g under normal conditions. In the high-nitrogen-treated plants, the nitrogen content of the leaves and roots was approximately 27 and 25 mg/g, respectively; in addition, the growth rate of the shoots increased by 10.2% compared with that of the control, but the new root biomass decreased by 37.8%. However, under the low-nitrogen treatment, the total nitrogen content decreased to 13 mg/g, the growth rate of aboveground shoots decreased by 33.6% and the root biomass increased significantly by approximately 86.5% compared with those of the control.

### Photosynthetic Rate and Chlorophyll Under Different Nitrogen Supplies

Nitrogen levels affect the chlorophyll content and net photosynthetic rate of crabapple. The content of chlorophyll a and chlorophyll b in mature leaves increased with increasing nitrogen application, and the pigment content in leaves significantly decreased in plants treated with low nitrogen. Specifically, chlorophyll a and chlorophyll b decreased by 31.2 and 33.8%, respectively, compared with those of the controls (Figure 1D). The change trend of Pn in mature leaves was similar to that of chlorophyll, the Pn increased with increasing nitrogen level, and the Pn decreased by 16.3% in leaves of plants treated with low nitrogen but slightly increased in leaves of plants treated with high nitrogen compared with that in the controls (Figure 1E).

### Changes in Carbohydrates Under Different Nitrogen Supplies

**FIGURE 2:**
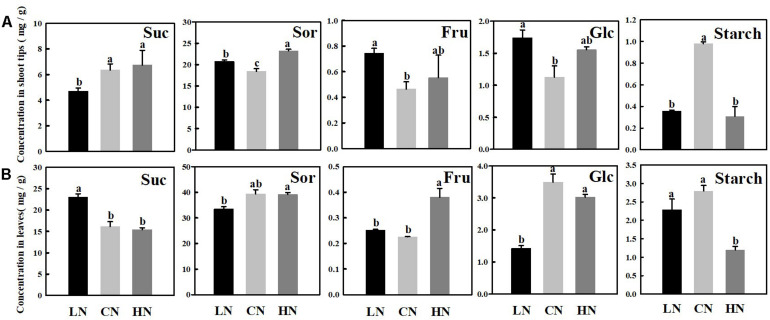
Changes in concentrations of sucrose (Suc), sorbitol (Sor), fructose (Fru), glucose (Glc), and starch in shoot tips **(A)** and leaves **(B)** treated with different nitrogen levels. LN means low nitrogen level contains 0.3 mM nitrogen; CN means common nitrogen level contains 6 mM nitrogen; HN means high nitrogen level contains 30 mM nitrogen. Each value is mean of three replicates ± SD. Bars labeled with different letters indicate significant difference between treatments.

The main soluble carbohydrates in the shoot tips (Figure 2A) and leaves (Figure 2B) of crabapple were sucrose, sorbitol, fructose, and glucose. Sucrose and sorbitol, the critical photosynthetic assimilates in apple plants, increased in the shoot tips treated with high nitrogen, unlike in leaves, both the contents of which were lower slightly than those of the controls in high-nitrogen conditions. Furthermore, under different nitrogen levels, the change in fructose content in the shoot tips and leaves showed a similar trend, which reflected an increase in content in both treatments, and the increase in fructose in the leaves was more apparent than that in shoot tips in high-nitrogen conditions. However, it is interesting to note that the glucose content in the leaves was lower than that in the controls in both treatments, whereas the change in the shoot tips was opposite. It is fairly obvious that the content of glucose decreased by approximately 59.3% in the leaves of plants treated with low nitrogen compared with that of the controls. The starch concentration in shoot tips was significantly decreased in both the low and high-nitrogen treatments compared with that of control. However, the significant change of starch in the leaves did not be caught in low-nitrogen supply condition.

### Expression of Genes Related to Sugar Metabolism Under Different Nitrogen Supplies

**FIGURE 3:**
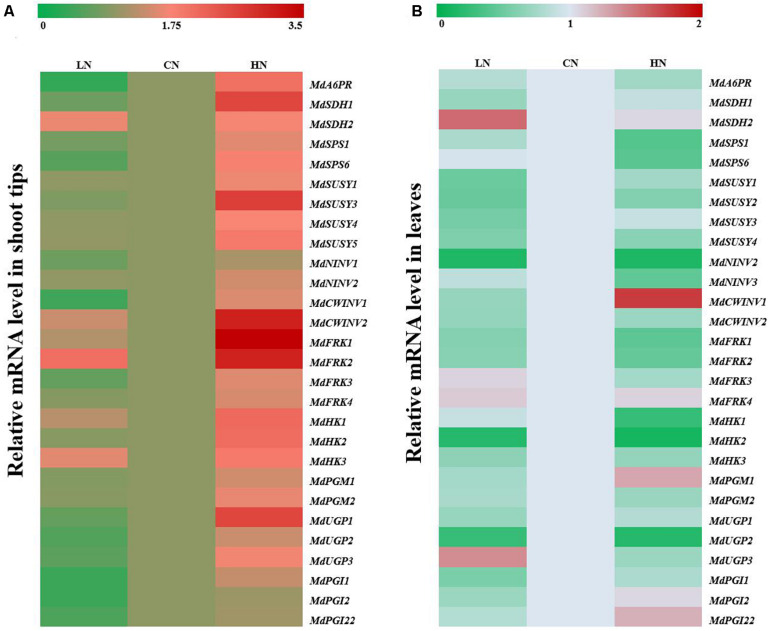
Relative mRNA expression of genes (*MdA6PR*, *MdSDHs*, *MdSPSs*, *MdSUSYs*, *MdNINVs*, *MdCWINVs, MdFRKs MdHKs, PGMs*, *PGIs*, and *UGPs*) encoding sugar metabolism in apple shoot tips **(A)** and leaves **(B)** treated with different nitrogen levels. LN means low nitrogen level contains 0.3 mM nitrogen; CN means common nitrogen level contains 6 mM nitrogen; HN means high nitrogen level contains 30 mM nitrogen. For each sample, transcript levels were normalized with those of *Actin*. Each value is mean of three independent replicates ± SD and the specific values are presented in Supplementary Table 1.

To further explore the influence of different nitrogen levels on sugar metabolism in the shoot tips and leaf cells of crabapple, the mRNA expression of the main genes was measured in samples of the three groups. *MdA6PR*, *MdSDH1* and *MdSDH2* are major genes involved in sorbitol metabolism. Increased application of nitrogen significantly enhanced the expression level of *MdA6PR* in the shoot tips, and the expression of *MdA6PR* under low-nitrogen conditions decreased approximately four times compared with that of the controls. The relative expression levels of *MdSDH1* and *MdSDH2* in the shoot tips prominently increased by 3 and 0.8 times compared with those of the controls, respectively, under high-nitrogen conditions. However, the expression of *MdSDH2* in the shoot tips increased by 1-fold compared with that of the controls, while the expression change of *MdSDH1* was inconspicuous under low-nitrogen conditions (Figure 3A). However, in the leaves of crabapple, *MdSDH1* expression was inhibited under both high and low nitrogen supply, especially under low-nitrogen conditions, and the expression level decreased by 31% compared with that of the controls. The change trend of *MdSDH1* expression was in accordance with that of *MdA6PR*, which is involved in the synthesis of sorbitol. In contrast, the expression of *MdSDH2* in the leaves was significantly up-regulated under low nitrogen supply, but no obvious change was observed under high nitrogen supply compared with that of the controls (Figure 3B).

*MdSPS*, *MdSUSY*, *MdNINV* and cell wall invertase *MdCWINV* play extremely important roles in sucrose metabolism. Expression of the sucrose phosphatase gene *MdSPS* in the shoot tips increased with increasing nitrogen content. Compared with those of the controls, the expression levels of *MdSPS1* and *MdSPS6* decreased by 17.3 and 38.3% under low-nitrogen conditions and increased by 58.6 and 81.4% under high-nitrogen conditions, respectively, which was consistent with the change trend of sucrose content in the shoot tips (Figure 2A). However, *MdSPS* expression in the leaves did not change significantly under low nitrogen supply and decreased by approximately 50% under high-nitrogen treatment compared with that of the controls (Figure 3B). Moreover, 4 *MdSUSY* genes in leaves showed a similar expression change trend; that is, the expression of *MdSUSY*s was inhibited under both high and low nitrogen supply, it was significantly up-regulated under high-nitrogen conditions in the shoot tips, and no significant expression change in *MdSUSY* was found under low nitrogen supply compared with that of the controls. The expression trends of *MdNINV*s and *MdCWINV2* were similar to those of *MdSUSY*s in the leaves; in particular, *MdNINV1* expression under high and low nitrogen supply was only 1/7 of that of the controls. For *CWINV*s, there was an opposite expression change between *MdCWINV1* and *MdCWINV2* in the leaves of plants treated with high nitrogen. Clear up-regulation of the expression of *MdCWINV1* was observed, and a significant decrease in the expression of *MdCWINV2* in the leaves was also observed (Figure 3B). However, the expression of both *MdNINV*s and *MdCWINV*s in the shoot tips showed an obvious up-regulation under high-nitrogen conditions.

With respect to the genes associated with hexose metabolism, compared with that in the controls, the expression of *MdFRK*s and *MdHK*s in the shoot tips of plants under high nitrogen supply increased to different degrees; among them, *the MdFRK1* and *MdHK2* expression levels were higher than others. Moreover, under low nitrogen supply, the expression of *MdFRK1* and *MdFRK4* was similar to that of the controls, *MdFRK2* was significantly up-regulated, and *MdFRK3* was slightly downregulated (Figure 3A). However, the expression of all *MdFRK*s and *MdHK*s in the leaves was inhibited under high nitrogen supply, except that of *MdFRK4*, which was not significantly affected by nitrogen content. Coinciding with the trends of *MdFRK1* and *MdFRK2* expression, the expression of *MdHK*s in the leaves of plants treated with high and low nitrogen was lower than that of the controls at some point. Among them, *MdHK2* expression decreased to less than 1/5 of that of the controls, and its expression abundance in the leaves was the highest among these three genes (Figure 3B). However, the expression differences of *MdHK*s in the shoot tips were relatively small under low nitrogen and normal nitrogen supplies. Compared with that in the controls, the expression of *MdHK1* and *MdHK3* in the low-nitrogen-treated shoot tips was slightly up-regulated, but there was no significant change in *MdHK2* expression (Figure 3A).

### Expression of Genes Related to Sugar Transport Under Different Nitrogen Supplies

**FIGURE 4:**
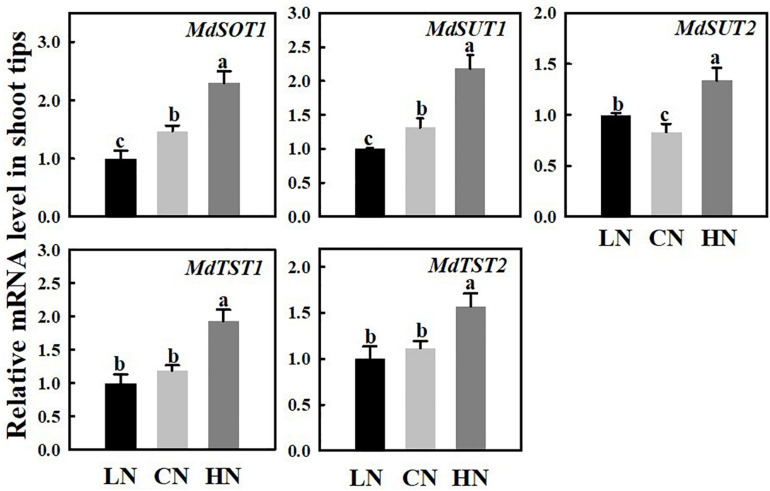
Relative mRNA expression for genes (*MdSOT1*, *MdSUTs*, and *MdTSTs*) encoding sugar transporters in shoot tips treated with different nitrogen levels. LN means low nitrogen level contains 0.3 mM nitrogen; CN means common nitrogen level contains 6 mM nitrogen; HN means high nitrogen level contains 30 mM nitrogen. For each sample, transcript levels were normalized with those of *Actin*. Each value is mean of three independent replicates ± SD. Bars labeled with different letters indicate significant difference between treatments.

The mRNA expression levels of the sugar transporters SOT, SUT, and TST in the shoot tips were measured under different nitrogen supply levels (Figure 4). The expressions of *MdSOT1*, *MdSUT1/2*, and *MdTST1/2* were approximately 1.56 times, 1.65/1.62 times, and 1.63/1.4 times those of the controls, respectively, compared with that of the controls under high nitrogen supply. However, under low-nitrogen conditions, the expression of *MdSOT1* and *MdSUT1* was downregulated, the expression of *MdSUT2* was slightly higher than that of the controls, and there were no obvious changes in the expression levels of *MdTST1* or *MdTST2* compared with those of the controls.

### Activity of Enzymes Related to Sugar Metabolism

**FIGURE 5:**
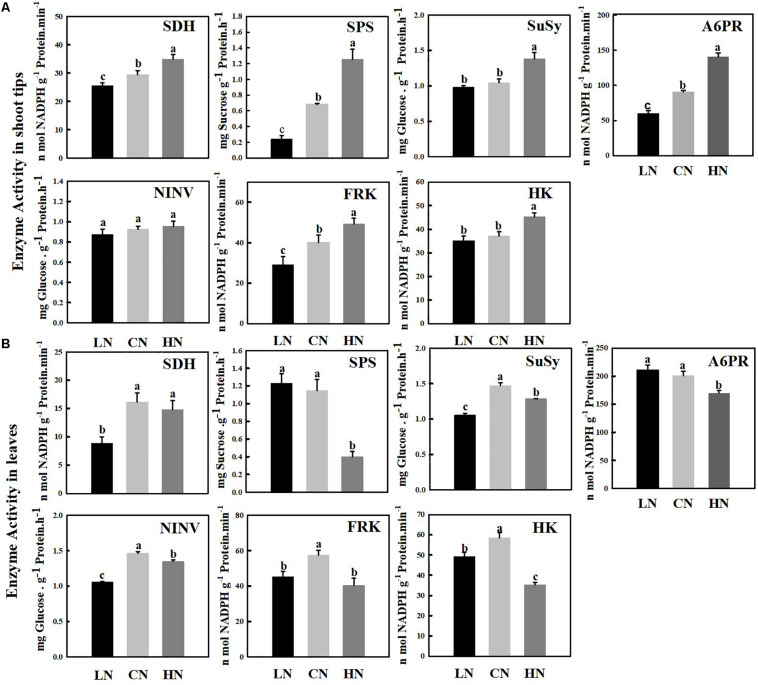
Changes in enzyme activities involved in sugar metabolism in shoot tips **(A)** and leaves **(B)** treated with different nitrogen levels. LN means low nitrogen level contains 0.3 mM nitrogen; CN means common nitrogen level contains 6 mM nitrogen; HN means high nitrogen level contains 30 mM nitrogen. Each Value is mean ± SD for three biological replicate samples. Bars labeled with different letters indicate significant difference between treatments.

The SDH, A6PR, SPS, SUSY, NINV, FRK, and HK enzymes, which are related to sugar metabolism, were measured in the shoot tips (Figure 5A) and leaves (Figure 5B) of crabapple. The activity of SDH, A6PR, SPS, and FRK in the shoot tips increased with increasing nitrogen application level, and the enzyme activity was the highest in the high-nitrogen conditions; the activities were approximately 1.84, 1.56, 1.19, and 1.23 times those of the controls, respectively. However, there were no visible changes in the activity of SUSY or HK between normal and low-nitrogen conditions, and both activities were significantly lower than those of shoot tips of plants treated with high nitrogen. In addition, the activity of SUSY and HK under high nitrogen supply was 1.32 and 1.22 times that of the controls, respectively. The change in nitrogen supply level did not cause a distinct change in NINV activity.

Sucrose phosphate synthase is the major rate-limiting enzyme during sucrose synthesis in plants, and its activity level was similar in the leaves of plants treated with low and normal nitrogen, but high nitrogen supply reduced its activity significantly to 1/3 of that of the control. The activities of SUSY, NINV, FRK, and HK reached their highest levels under normal nitrogen supply, and a significant increase and decrease in nitrogen supply reduced the activity of these enzymes in the leaves; however, the activity of SDH did not decrease significantly under high nitrogen supply. The enzyme activity of SUSY, NINV, and SDH reached the lowest level in the leaves of plants treated with low nitrogen approximately 0.55–0.71 times that of the level of the controls.

## Discussion

The most direct apparent way by which nitrogen affects plant growth and development is through changes in biomass; an adequate nitrogen supply provides a sufficient material basis for the synthesis of proteins and structural carbohydrates, resulting in rapid cell division, rapid growth and increased biomass (Zhu et al., 2004; Dash et al., 2015; Huang et al., 2018). In the present study, the enhanced net photosynthetic rate (Pn) of leaves resulted in an increase in the accumulation of photosynthetic metabolites such as sorbitol under high nitrogen supply, and an increase of the shoot tip growth rate, while a significant decrease of the root biomass (Figure 1). With the increase of nitrogen application, the expression level of *MdSPS* was significantly down-regulated after up-regulation, the sucrose content in leaves was significantly reduced. On the contrary, the obvious accumulation of sugar content was observed in the shoot tips, which led to the corresponding rapid growth of the shoot tips (Figure 2). The results of this study indicated that crabapple plants preferentially allocated more photosynthetic assimilates to fast-growing shoot tip tissues under high nitrogen supply.

The role of nitrogen in promoting shoot tips growth is closely related to the pattern of carbohydrate accumulation and distribution in plants (Huppe and Turpin, 1994; Scheible et al., 1997; Druege et al., 2000). Different nitrogen supply level affected the synthesis, metabolism and transport of carbohydrates in crabapple different tissues. After analyzing the data concerning crabapple plants treated with various nitrogen concentrations, we found that the expressions of *MdA6PR* and *MdSPS* were up-regulated under high nitrogen conditions (Figure 3), which promoted the synthesis of sorbitol and sucrose in the stem tips (Figure 2A). This result suggested that more photosynthetic assimilates as energy substances or signaling molecules were transported from source leaves to the sink to participate in shoot tip growth under high nitrogen conditions. Interestingly, the expression of *MdSPS* in the shoot tips at low nitrogen supply was not obviously different from that at a normal nitrogen supply, whereas *MdA6PR* expression decreased significantly. These data suggested that a normal nitrogen supply would prioritize the improvement of the relative synthesis ability of sorbitol rather than sucrose, which was also consistent with the sorbitol and sucrose content we measured (Figure 2A). In addition, sorbitol and sucrose are the major forms of carbohydrate transport over long distances in the Rosaceae plants (Rennie and Turgeon, 2009; Eom et al., 2012; Li et al., 2018). The timely unloading and utilization of carbohydrate in the sink is conducive to the formation of a concentration gradient between the source leaves and the stem tips to drive the continuous transportation of sorbitol and sucrose to shoot tips, which plays a vital role in regulating the distribution of photosynthetic assimilates and the sink strength (Grechi et al., 2007). *MdSOT*, *MdSUT* and *MdTMT* are involved in the unloading of sorbitol and sucrose, and the increase of their expression levels is conducive to the delivery of sorbitol and sucrose from the extracellular space into cells (Li et al., 2012; Wei et al., 2014; Figure 4). Therefore, the accumulation of sorbitol and other sugars in shoot tips under high nitrogen conditions may be related to the enhancement of carbohydrate unloading capacity.

Plant leaves synthesize photosynthetic assimilates and transport them to the sink organs over long distances in the phloem. Carbohydrate accumulation in the sink organs accelerates C and N metabolism in plants (Li et al., 2012). Sorbitol and sucrose accumulated in apple shoot tips under high nitrogen condition provided sufficient material and energy for rapid growth and development of shoot tips. In response to massive accumulation of sorbitol and sucrose, the gene expression levels (Figure 3A) of *MdSDH* and *MdSUSY*, both of which severely break down sorbitol and sucrose, were also elevated in the shoot tips of plants under high nitrogen supply. SUSY is considered to be a marker of sink strength (Coleman et al., 2009). Under high nitrogen conditions, the activity of SUSY, like SDH, was significantly increased, while the activity of NINV was not significantly changed (Figure 5A), indicating that SUSY, rather than NINV, played a role in increasing sink strength of shoot tips under high nitrogen conditions. High nitrogen supply not only promoted the synthesis but also improved the degradation rates of sorbitol and sucrose compared with that of the controls. These results are in accordance with previous findings concerning transgenic apple leaves overexpressing *MdFK2* (Yang et al., 2018). Additionally, the degradation products of sorbitol and sucrose are fructose and glucose, which are able to be phosphorylated by FRK and HK, respectively, for entry into glycolysis and the tricarboxylic acid cycle and for provision of energy for plant growth and development (Zhu et al., 1997; Li et al., 2016). The results of this study showed that hexose phosphorylation in the shoot tips was also promoted due to the activity of the main enzymes (Figure 5A) FRK and HK, and the expression of corresponding genes (Figure 3A) involved in hexose metabolism increased to some extent. These results showed that the improved nitrogen level both increased sugar synthesis and metabolic utilization capacity in the shoot tips and accelerated the carbon flow to meet the demand for sugar needed for shoot growth.

Studies have shown that the conversion between F6P, G6P, G1P, and UDP-glucose is a reversible reaction catalyzed by PGM, PGI, and UGP (Li et al., 2012). High nitrogen supply accelerated the degradation of sucrose and accumulated a large amount of UDP-glucose in shoot tip. At the same time, the expression of UDP, which catalyzed G6P to form UDPG, was up-regulated, leading to the accumulation of a large amount of UDP-glucose in shoot tip cells under high nitrogen supply (Figure 3). While UDP-glucose is an important component of cellulose-synthesis in cell wall of shoot tips. Therefore, the accumulation of UDP-glucose provided a material basis for the rapid division and elongation of shoot tip cells. In plant cells, PGM involved in photosynthesis, respiration and synthesis of cell walls, and PGM catalytic the conversion between intermediate G1P and G6P in the process of sucrose metabolism, While G6P enters the pentose phosphate pathway to produce a large amount of NADPH, which participates in a variety of metabolic reactions as a hydrogen donor (Liu et al., 2012; Dussert et al., 2013). High nitrogen supply resulted in up-regulation of *MdPGM* expression, but no significant change in *MdPGI* expression in shoot tips (Figure 3), which indicated that more G1P may be converted to G6P and then enter the pentose phosphate pathway at high nitrogen supply, while F6P is more likely to enter the glycolysis and TCA cycles to produce energy or intermediates for other processes and then accelerate metabolic reactions due to the low conversion efficiency between G6P and F6P.

## Conclusion

**FIGURE 6:**
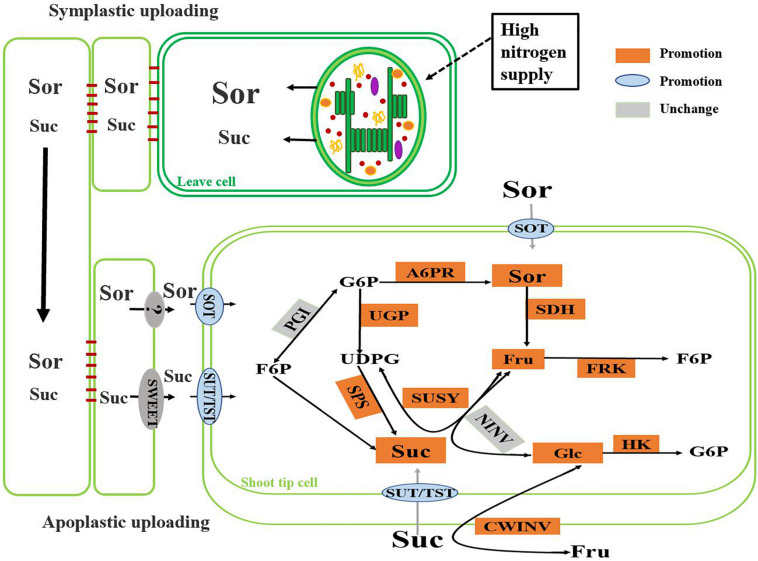
Change of main sugar metabolism pathway in crabapple shoot tips under high nitrogen supply.

In our experiments, under high nitrogen supply, the crabapple plants preferentially allocated more photosynthetic assimilates to the growth point of stem tips. The up-regulated expressions of *MdSOT*, *MdSUT*, and *MdTST* improved the unloading capacity of carbohydrate in the shoot tips, and the increase of the activities of A6PR and SPS in the shoot tips also significantly enhanced the sink strength. High nitrogen supply accelerated the synthesis of sorbitol and sucrose in shoot tip, as well as their decomposition. Increased activity of SDH, SUSY led to higher levels of glucose and fructose in the cells, which were further phosphorylated to produce energy to supply rapid growth of the shoot tips (Figure 6). It is worth noting that under high nitrogen supply, some of the main sugar levels in source leaves, including sorbitol, sucrose, and glucose, slightly decreased, which may be associated with assimilate competition due to the rapid growth of the shoot tips. In addition, the use of redundant nitrogen also requires plants to exhibit increased respiration, and this process also consumes carbohydrates. This altered sugar metabolism combined with related changes in the sugar transport system greatly benefits the regulation of carbohydrate osmotic adjustment and the adaptability of plants to nitrogen levels.

## Data Availability Statement

The original contributions presented in the study are included in the article/Supplementary Material, further inquiries can be directed to the corresponding author/s.

## Author Contributions

LZ and SS performed the majority of experiments. YL and BL performed the essential experiments. SM analyzed and discussed data. BM and ML supervised the work. All authors contributed to final manuscript.

## Conflict of Interest

The authors declare that the research was conducted in the absence of any commercial or financial relationships that could be construed as a potential conflict of interest.

## Abbreviations

N: nitrogen
SUTs: sucrose transporters
A6PR: aldose-6-phosphate reductase
AINV: vacuolar acid invertase
CWINV: cell wall invertase
DTT: dithiothreitol
FRK: fructokinase
Fru: fructose
F6P: fructose-6-phosphate
Glc: glucose
HK: hexokinase
NINV: neutral invertase
PVPP: polyvinylpolypyrrolidone
SDH: sorbitol dehydrogenase
Suc: sucrose
SUSY: sucrose synthase
SPS: sucrose phosphate synthase
SPP: sucrose-phosphate phosphatase
UDPG: UDP-glucose
MSTFA: N-methyl -N-trimethylsilyl-trifluoroace-tamide
G6PDH: G6P dehydrogenase
AEBSF: 4-(2-amino) benzenesulfonyl fluoride hydrochloride
PGI: phosphoglucoisomerase.
