# Supplementary material for: Nitrogen Levels Regulate Sugar Metabolism and Transport in the Shoot Tips of Crabapple Plants
Source: Front Plant Sci. 2021 Mar 10;12:626149. doi: 10.3389/fpls.2021.626149 (PMC7988234; doi:10.3389/fpls.2021.626149)
Supplement: Supplementary file 1 [file Table_1.docx]

Supplementary Table 1. Relative mRNA level of genes in shoot tips and leaves

| Gene |  | Relative mRNA level in shoot tips | | |  | Relative mRNA level in leaves | | |  |
| --- | --- | --- | --- | --- | --- | --- | --- | --- | --- |
|  |  | LN | CN | HN |  | LN | CN | HN |  |
| *MdA6PR* |  | 0.36±0.01c | 1±0.17b | 2.04±0.09a |  | 0.82±0.01b | 1±0.03a | 0.73±0.02c |  |
| *MdSDH1* |  | 0.77±0.22b | 1±0.17b | 2.59±0.12a |  | 0.69±0.01c | 1±0.03a | 0.88±0.03b |  |
| *MdSDH2* |  | 1.65±0.03a | 1±0.10b | 1.73±0.16a |  | 1.53±0.01a | 1±0.04b | 0.88±0.03b |  |
| *MdSPS1* |  | 0.83±0.02b | 1±0.17b | 1.59±0.07a |  | 0.78±0.11b | 1±0.02a | 0.39±0.01c |  |
| *MdSPS6* |  | 0.62±0.02c | 1±0.17b | 1.81±0.08a |  | 0.97±0.01a | 1±0.03a | 0.41±0.01b |  |
| *MdSUSY1* |  | 1.02±0.03b | 1±0.17b | 1.67±0.08a |  | 0.49±0.01c | 1±0.15a | 0.74±0.02b |  |
| *MdSUSY2* |  | — | — | — |  | 0.48±0.01c | 1±0.03a | 0.59±0.02b |  |
| *MdSUSY3* |  | 0.90±0.02b | 1±0.17b | 2.68±0.12a |  | 0.53±0.01c | 1±0.03a | 0.90±0.03b |  |
| *MdSUSY4* |  | 1.02±0.03b | 1±0.17b | 1.76±0.08a |  | 0.58±0.01b | 1±0.05a | 0.90±0.03b |  |
| *MdSUSY5* |  | 1.04±0.03b | 1±0.17b | 1.90±0.09a |  | — | — | — |  |
| *MdNINV1* |  | 0.77±0.10b | 1±0.07ab | 1.2±0.11a |  | — | — | — |  |
| *MdNINV2* |  | 1.02±0.02b | 1±0.10b | 1.46±0.13a |  | 0.15±0.02b | 1±0.04a | 0.14±0.01b |  |
| *MdNINV3* |  | — | — | — |  | 0.86±0.10a | 1±0.04a | 0.44±0.03b |  |
| *MdCWINV1* |  | 0.46±0.01c | 1±0.10b | 1.53±0.14a |  | 0.68±0.08c | 1±0.04b | 1.74±0.13a |  |
| *MdCWINV2* |  | 1.42±0.19b | 1±0.11c | 3.08±0.09a |  | 0.68±0.01b | 1±0.08a | 0.70±0.01b |  |
| *MdFRK1* |  | 1.25±0.17b | 1±0.11b | 3.49±0.10a |  | 0.61±0.07b | 1±0.04a | 0.43±0.03c |  |
| *MdFRK2* |  | 2.04±0.27b | 1±0.11c | 3.07±0.09a |  | 0.62±0.01b | 1±0.08a | 0.46±0.12c |  |
| *MdFRK3* |  | 0.71±0.01c | 1±0.10b | 1.56±0.14a |  | 1.09±0.13a | 1±0.04ab | 0.75±0.06b |  |
| *MdFRK4* |  | 0.94±0.02b | 1±0.10b | 1.51±0.14a |  | 1.12±0.13a | 1±0.04a | 1.09±0.08a |  |
| *MdHK1* |  | 1.28±0.17b | 1±0.11b | 2.12±0.06a |  | 0.90±0.01a | 1±0.08a | 0.25±0.01b |  |
| *MdHK2* |  | 0.96±0.13b | 1±0.11b | 2.07±0.06a |  | 0.17±0.01b | 1±0.08a | 0.1±0.01c |  |
| *MdHK3* |  | 1.58±0.21a | 1±0.11b | 1.90±0.05a |  | 0.65±0.01b | 1±0.08a | 0.68±0.01b |  |
| *MdPGM1* |  | 0.93±0.13b | 1±0.07b | 1.46±0.13a |  | 0.75±0.01c | 1±0.08b | 1.28±0.01a |  |
| *MdPGM2* |  | 0.96±0.13b | 1±0.07b | 1.64±0.15a |  | 0.77±0.01b | 1±0.08a | 0.70±0.08b |  |
| *MdUGP1* |  | 0.71±0.10c | 1±0.07b | 2.58±0.23a |  | 0.69±0.01c | 1±0.08a | 0.81±0.01b |  |
| *MdUGP2* |  | 0.58±0.08c | 1±0.07b | 1.41±0.13a |  | 0.25±0.01b | 1±0.01a | 0.17±0.01c |  |
| *MdUGP3* |  | 0.66±0.09c | 1±0.07b | 1.70±0.15a |  | 1.39±0.01a | 1±0.08b | 0.71±0.01c |  |
| *MdPGI1* |  | 0.42±0.08c | 1±0.04b | 1.38±0.04a |  | 0.56±0.04c | 1±0.02a | 0.79±0.02b |  |
| *MdPGI2* |  | 0.42±0.08b | 1±0.04a | 1.10±0.03a |  | 0.71±0.06b | 1±0.02a | 1.07±0.03a |  |
| *MdPGI22* |  | 0.55±0.10c | 1±0.04b | 1.12±0.03a |  | 0.82±0.06c | 1±0.02b | 1.24±0.03a |  |

Relative mRNA expression of genes (*MdA6PR*, *MdSDHs*, *MdSPSs*, *MdSUSYs*, *MdNINVs*, *MdCWINVs*, *MdFRKs*, *MdHKs*, *PGMs*, *PGIs* and *UGPs*) encoding sugar metabolism in apple shoot tips and leaves treated with different nitrogen levels. LN means low nitrogen level contains 0.3 mM nitrogen; CN means common nitrogen level contains 6m M nitrogen; HN means high nitrogen level contains 30 mM nitrogen. For each sample, transcript levels were normalized with those of Actin. Each value is mean of three independent replicates ±SD. The different letters mean significant different at p < 0.05.
